# *Cheiriphotis trifurcata*, new species (Crustacea, Amphipoda, Corophiidae, Protomedeiinae) from the Seagrass Bed of the Lower Gulf of Thailand

**DOI:** 10.3897/zookeys.187.3219

**Published:** 2012-04-27

**Authors:** K. Wongkamhaeng, B.A.R. Azman, R. Puttapreecha

**Affiliations:** 1 Marine and Coastal Resources Institute (MACORIN), Prince of Songkla University, 90112 THAILAND; 2 Marine Ecosystem Research Centre (EKOMAR), Faculty of Science and Technology, Universiti Kebangsaan Malaysia, 43600 Bangi, Selangor, MALAYSIA; 3 Southern Marine and Coastal Resources Research Center, 158 Moo 8, Phawong, Muang, Songkhla 90100 THAILAND

**Keywords:** Crustacea, Amphipoda, Isaeidae, *Cheiriphotis trifurcata*, Gulf of Thailand, taxonomy

## Abstract

A new species of corophiid Amphipoda, *Cheiriphotis trifurcata*, collected from the seagrass bed of the Lower Gulf of Thailand, is described. *Cheiriphotis trifurcata* is characterized by its trifurcated tip of the modified setae on the outer ramus in male pleopod 3. In this paper, the new species is fully described and compared with related species and a complete key of the 16 valid species in the genus *Cheiriphotis* is given.

## Introduction

Species of the genus *Cheiriphotis* Walker, 1904, are predominant and widespread in both marine circumtropical and warm-temperate waters of the world. Of the 15 valid species that have been described so far, only *Cheiriphotis megacheles* (Giles, 1885) was reported in the Andaman Sea while the Gulf of Thailand has no reports of amphipods in this genus (e.g. Angsupanich and Kuwabara 1995; Angsupanich et al. 2005; Ariyama et al. 2010; Bussarawich 1985; Chilton 1925; and Ruensirikul et al. 2007).

*Cheiriphotis megacheles* was first described from the Bay of Bengal in 1885 by Giles, and later reported from Sri Lanka (Ceylon) by Walker in 1904. However, the specimens from the two localities are clearly distinctive in male gnathopod 1 and uropod 3 which Walker (1904) concluded as an ontogeny. Salman and Jabbar (1990) redescribed *Cheiriphotis megacheles* based on material collected from the north-west of the Arabian Gulf and found that the variation between Giles’s and Walkers’ specimens is not an ontogeny, instead they are two distinct species.In the present study we provide a detailed description and illustration of both male and female species of *Cheiriphotis trifurcata* new species collected from the seagrass bed area. This description represents the first record of the genus *Cheiriphotis* in the Gulf of Thailand. A key for the genus *Cheiriphotis* is also presented.

## Material and methods

**Figure 1.**
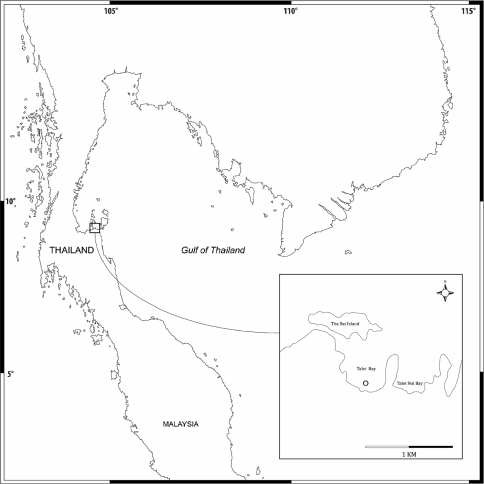
Map of sampling area

Amphipods were collected using a 20×20 cm^2^ Ekman’s grab in a seagrass bed of Talet bay (Figure 1). The sites were visited at low tide and amphipods were collected from the subtidal zone. Seagrass and sediment were sieved with a 0.5 mm sieve. Amphipod specimens were sorted out and fixed in formalin for 1 week and then stored in 70% alcohol. In the laboratory, the animals were examined using a compound microscope and later selected for dissection. The appendages were examined and ﬁgures were produced using an Olympus CH30 light microscope with a camera lucida. The following abbreviations are used: A, antenna; G, gnathopod; HD, head; LL, lower lip; MD, mandible; MX, maxilla; MP, maxilliped; P, pereopod; Pl, pleopod; T, telson; U, uropod; UR, urosome; UL, upper lip; r, right; l, left; ♂, male; ♀, female. The type material of the new species is deposited at Prince of Songkla University Zoological Collection (PSUZC) and the Universiti Kebangsaan Malaysia Muzium Zoologi (UKMMZ), Malaysia.

## Results

**Corophiidae Leach, 1814**

**Protomedeiinae Myers & Lowry, 2003**

### Cheiriphotis

(Giles, 1885)

http://species-id.net/wiki/Cheiriphotis

#### Type species.

*Melita megacheles* Giles, 1885 by monotypy.

#### Diagnosis.

Eyes small. Antenna 1, accessory flagellum pluriarticulate. Mandibular palp article 3 rectolinear or clavate. Coxae small, relatively short, coxa 1 dilated, produced forward. Gnathopod 1 (male) subchelate. Gnathopod 2 subchelate and greatly larger than gnathopod 1. Pereopods 6–7, dactylus elongate, falcate. Uropods 1 – 2 biramous; rami slighty subequal; peduncle with ventrodistal process. Uropod 3 uniramous. Telson entire.

#### Species composition.

*Cheiriphotis* contains 16 species: *Cheiriphotis australiae* Stebbing, 1910; *Cheiriphotis delloyei* Pirlot, 1934; *Cheiriphotis durbanensis* K.H. Bamard, 1916; *Cheiriphotis erythraeus* Ruffo, 1969; *Cheiriphotis geniculata* K.H. Barnard, 1916; *Cheiriphotis madagascarensis* Ledoyer, 1979; *Cheiriphotis mediterranea* Myers, 1983; *Cheiriphotis megacheles* (Giles, 1885); *Cheiriphotis minima* Ledoyer, 1982; *Cheiriphotis neotropicalis* Valerio-Berardo, 2007; *Cheiriphotis pediformis* Myers, 1995; *Cheiriphotis quadrichelatus* Ortiz & Lalana, 1997; *Cheiriphotis rotui* Myers, 1989; *Cheiriphotis walkeri* Stebbing, 1918; *Cheiriphotis williamsoni* Salman & Jabbar, 1990;

### Cheiriphotis trifurcata sp. n.

urn:lsid:zoobank.org:act:37DAB18F-70EF-42C3-BD57-0E0911C11128

http://species-id.net/wiki/Cheiriphotis_trifurcata

#### Type material.

*Holotype*. ♂, THAILAND, Lower Gulf of Thailand, Talet Bay (09˚18'39.5"N, 99˚46'46.4"E), seagrass bed (associated with *Thalassia hemprichii*), 24 September 2008, Puttapreecha, R., PSUZC-CR-0264.

*Allotype*. ♀, collected with holotype, PSUZC-CR-0265 (adult female, 4.16 mm)

*Other material*. Same data as for holotype, UKMMZ-1446 (5♂; 15♀); PSUZC-CR-0266 (5♂; 20♀)

#### Description.

**Male** (*holotype*). Total body length 3.5 mm (from tip of rostrum to apex of telson). *Body* rather slender and subcylindrical. *Head* subequal in length to first 2 pereonites; rostrum not developed; inferior antennal sinus short and concave, about 0.3 times of head length; *eye* distinct. *Antenna 1* slightly longer than antenna 2, ratio of peduncular article 1–3 as 5:9:8; article 1 slender, with 2 postero-marginal setae; flagellum with 10 articles, 0.7 times as long as peduncle; accessory flagellum with 4 articles, last article scale-like. *Antenna 2* peduncle slender; article 1–4 in ratio of 2:5:4:2; inner margin of article 4 and 5 with long postero-marginal setae; article 5 shorter than 4; flagellum short with long setae, subeaqual in length to peduncular article 5, composed of 7 articles, last article scale-like.

*Upper lip* or labrum round and broad, with small depression in the middle and pubescent on each lobe. *Lower lip* inner lobe small and pubescent, mandibular process well developed; outer plate with a group of finger-like setae on the inner face of the outer lobe, covered with thin hair-like setae. *Mandible*, both incisors with 5 teeth; lacinia mobilis armed with 4 teeth on the left side and 5 teeth on the right side; molar process columnar, ridged distally; palp 3-articulate with ratios of 1:3:3, article 1 with 2 marginal setae, article 2–3 with apical and marginal setae. *Maxilla 1*, inner plate small with 2 apical setae, outer plate with 8 apical and marginal serrate robust setae; palp extending beyond outer plate, biarticulate with 6 apical serrate robust setae. *Maxilla 2*, inner plate with 19 slender marginal setae; outer plate larger than inner plate with 20 slender setae. *Maxilliped*, inner plate broad and short, reaching half of outer plate, apically provided with 4 conate setae and fine setae; outer plate broad, almost reaching palp article 2 with 7 conate setae; palp 4-articulate with ratio of 3:5:2:1.

**Pereon.** *Gnathopod 1* subchelate, smaller than gnathopod 2; coxal plate subtriangular, produced anteriorly with long fine setae on anteroventral corner; length ratio of articles from basis to dactylus about 14:5:6:13:10:9; basis slender, broader distally, posterior margin bearing long setae; ischium short, subrectangular with apical setae; merus subtriangular with posteromarginal setae, longer than ischium; carpus longer than propodus with plumose setae on posterior margin; propodus shorter than dactylus, palm oblique with a robust seta at the proximal half, surface of palm toothed; dactylus slightly longer than palm, falcate, inner margin with a robust seta,. *Gnathopod 2* subchelate; coxal plate short and wide, subrectangular, length ratio of articles from basis to dactylus about 9:5:8:8:19:19; basis robust, nearly as long as wide, broader distally, anterior margin straight, both sides naked; ischium subrectangular; merus longer than ischium; carpus distal and anterior margin fused with propodus; propodus enlarged, as long as wide, anterior margin with a row of plumose setae, posterior margin with short setae; palm transverse, with 4 blunt teeth and one acute palmar corner; dactylus slightly longer than palmar margin, inner margin smooth.

*Pereopod 3* slender and elongate; coxal plate small and suboval, with 3 plumose setae on anterior side; length ratio of articles from basis to dactylus 10:3:6:2:6:4; basis slender, distally extended; ischium short, subrectangular; merus longer than carpus, slightly produced anterodistally; carpus subrectangular, medially broad, posterior margin setose; propodus subrectangular; basis – propodus bearing plumose setae on both sides; dactylus falcate, long and thin, shorter than propodus. *Pereopod 4* rather similar to pereopod 3, coxal plate suboval with plumose setae on ventral side; length ratio of articles from basis to dactylus about 10:2:5:3:6:4; basis slender; ischium short, subrectangular; merus longer than carpus, slightly produced anterodistally; carpus subquadrate, shorter than propodus; basis to propodus with plumose setae on both margins; propodus long and narrow; dactylus long and thin, shorter than propodus. *Pereopod 5* shorter than pereopod 6 and 7; coxa bilobed; length ratio of articles from basis to dactylus about 14:2:3:3:6:3; basis subrectangular with plumose setae on both margins; ischium shortest with posteromarginal plumose setae; merus subequal to carpus, with posteromarginal plumose setae and 1 anterodistal seta; carpus with posteromarginal plumose setae; propodus with 4 robust setae along posterior margin; dactylus short, strongly curved. *Pereopod 6* elongate, 1.5 times as long as pereopod 5; coxa posteriorly produced with rounded lobe; length ratio of articles from basis to dactylus about 5:2:3:3:5:2; basis oval with plumose setae on both margins; ischium short with plumose setae on anteroventral corner; merus oblong, with plumose setae on both margins; carpus shorter than propodus, bearing long setae; propodus slender with marginal robust setae and setose posterodistally; dactylus falcate. *Pereopod 7* elongate, 1.6 times as long as pereopod 5; coxa short and wide, subtriangular, anteriorly produced; length ratio of articles from basis to dactylus about 13:5:7:7:11:6; basis posteriorly produced, bearing plumose setae on both margins; ischium short and subquadrate with plumose setae on anterodistal corner; merus elongate with plumose setae on both sides; carpus subequal to merus, both margins with sparse setae; propodus slender, longer than merus, distally extended; bearing setae on both margins and one robust seta on anterodistal corner; dactylus falcate, with one thin seta at 2/3 from proximal end.

**Pleon.** *Pleopods 1–2* well developed; peduncles subcylindrical, longer than broad and fringed with several plumose setae and a pair of retinaculae on the inner margin; inner ramus subequal to peduncle with 9–10 articles, outer ramus shorter than inner ramus, both rami with facial setae.

*Pleopod 3* similar to pleopod 1 and 2 except the tip of outer ramus modified; bearing long setae with sparse setule and having three additional modified setae with three forked tips respectively, outer ramus longer than inner ramus.

*Uropod 1* longest, extending beyond uropods 3; peduncle longer than both rami, beset with robust setae, peduncular apex bearing 3 posteroventral robust setae; outer and inner margins of both rami lined with a row of robust setae, distal margin rounded and bearing several robust setae. *Uropod 2* not reaching uropod 3, peduncle shorter than rami, both outer and inner margins with a row of robust setae; outer ramus slightly longer than inner one, both rami lined with a row of robust setae and distal margin bearing several short and long robust setae. *Uropod 3* uniramous, peduncle extended with robust seta on apex, subequal to ramus; apically 3 robust setae and 2 setae. *Telson* subtrapezoidal, distally excavated with long simple setae near both distal corners.

**Female.** (*allotype*). Total body length 4.2 mm (from tip of rostrum to apex of telson). – (sexually dimorphic characters).

*Antenna 1* flagellum with 12 articles.

**Pereon.** *Gnathopod 2* subchelate, smaller than that of male, basis to propodus setose; basis more slender, about 2.3 times as long as broad; carpus subtriangular, as long as broad; propodus suboval, longer than carpus, palm oblique and defined by a large bifid robust seta, palmar margin convex, distal end covered with sparse setae; dactylus curved with 5 inner marginal short setae. Coxa 4 and 5 longer than those of male.

*Pleopod 3* without modified tip of outer ramus.

**Figure 2A.**
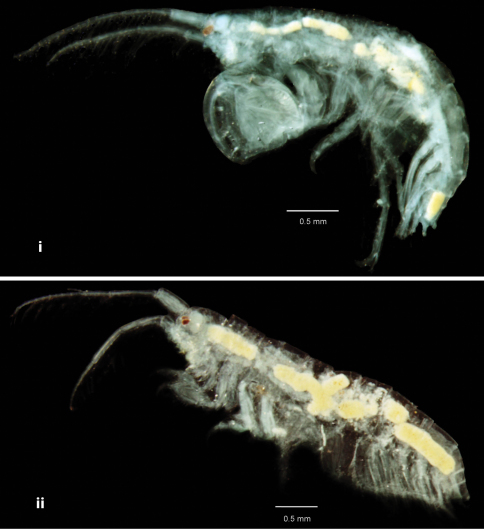
Photography of *Cheiriphotis trifurcata* sp. n. **i** holotype, male, (PSUZC-CR-0264), 3.47 mm. **ii** allotype, female, (PSUZC-CR-0265), 4.16 mm. Talet Bay, Lower Gulf of Thailand.

**Figure 2B.**
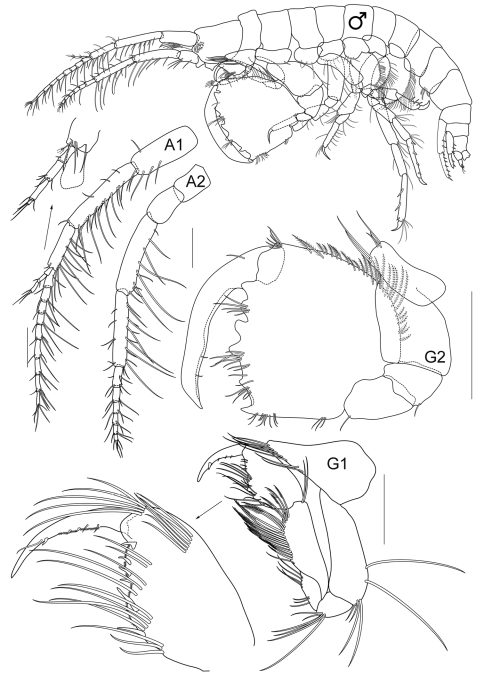
*Cheiriphotis trifurcata* sp. n., holotype, male, (PSUZC-CR-0264), 3.47 mm. Talet Bay, Lower Gulf of Thailand. All scales represent 0.2 mm.

**Figure 2C.**
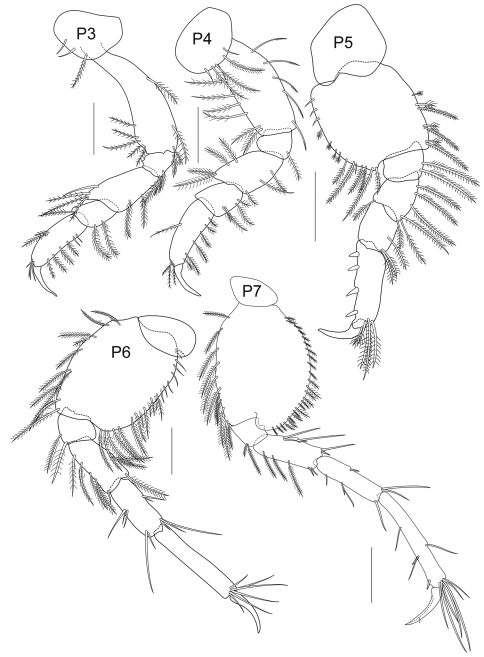
*Cheiriphotis trifurcata* sp. n., holotype, male, (PSUZC-CR-0264), 3.47 mm. Talet Bay, Lower Gulf of Thailand. All scales represent 0.2 mm.

**Figure 2D.**
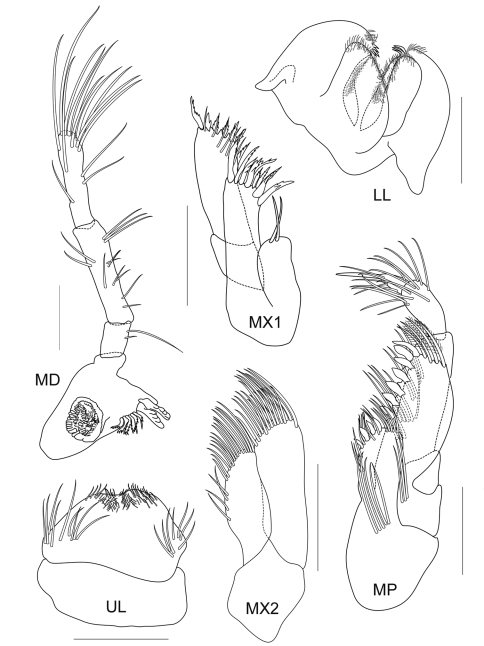
*Cheiriphotis trifurcata* sp. n., holotype, male, (PSUZC-CR-0264), 3.47 mm. Talet Bay, Lower Gulf of Thailand. All scales represent 0.1 mm.

**Figure 2E.**
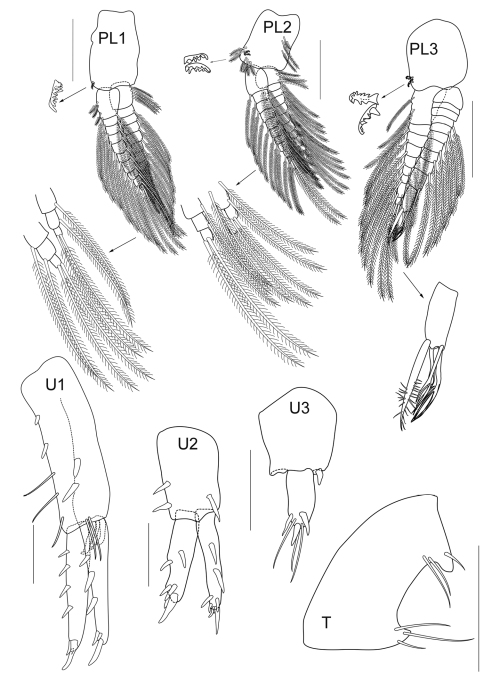
*Cheiriphotis trifurcata* sp. n., holotype, male, (PSUZC-CR-0264), 3.47 mm. Talet Bay, Lower Gulf of Thailand. Scales for U1– U3 and T represent 0.1 mm; PL1 – PL3 represent 0.2 mm.

**Figure 3A.**
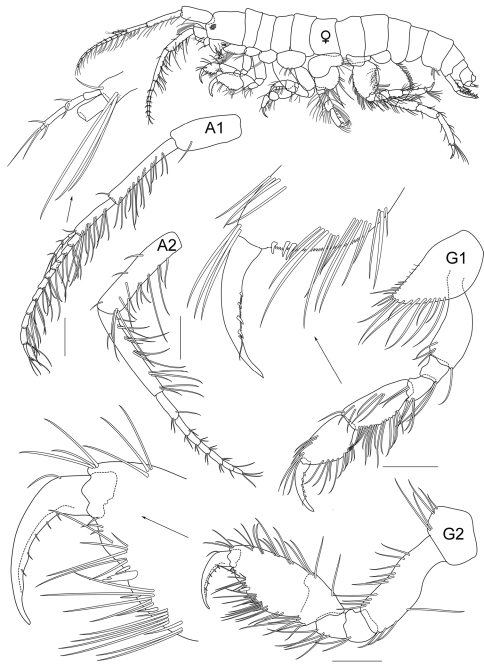
*Cheiriphotis trifurcata* sp. n., allotype, female, (PSUZC-CR-0265), 4.16 mm. Talet Bay, Lower Gulf of Thailand. All scales represent 0.2 mm.

**Figure 3B.**
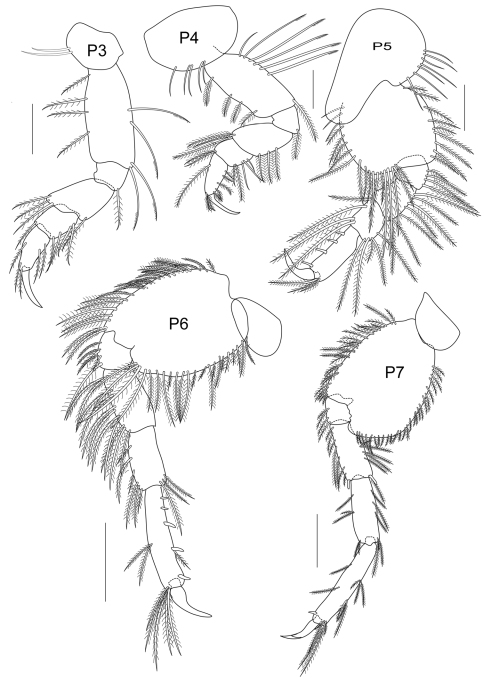
*Cheiriphotis trifurcata* sp. n., allotype, female, (PSUZC-CR-0265), 4.16 mm. Talet Bay, Lower Gulf of Thailand. All scales represent 0.1 mm.

**Figure 3C.**
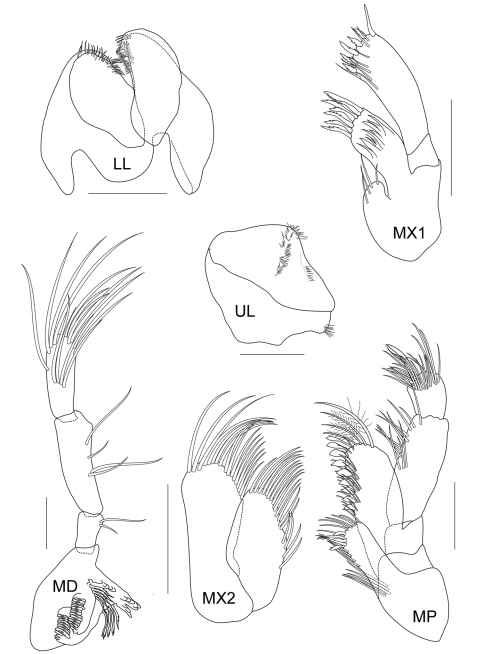
*Cheiriphotis trifurcata* sp. n., allotype, female, (PSUZC-CR-0265), 4.16 mm. Talet Bay, Lower Gulf of Thailand. All scales represent 0.1 mm.

**Figure 3D.**
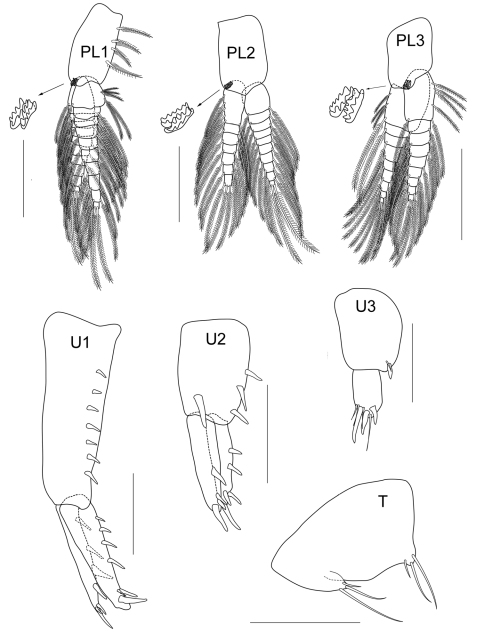
*Cheiriphotis trifurcata* sp. n., allotype, female, (PSUZC-CR-0265), 4.16 mm. Talet Bay, Lower Gulf of Thailand. Scale for U1-U3 and T represents 0.1 mm; remaining represents 0.2 mm.

#### Etymology.

The specific name “*trifurcata*” is from latin ‘tri = three’ and ‘furcated = forked’, referring to the distinct three forked tips of the modified setae on the outer ramus in male pleopod 3.

#### Remarks.

Even *Cheiriphotis trifurcata* shows a distinct character, with the presence of the three additional modified setae in male pleopod 3 and each seta equipped with three forked tips, but this character might be overlooked in other species. Besides, the general characters in the present species are closely related to *Cheiriphotis williamsoni*, *Cheiriphotis neotropicalis*, *Cheiriphotis mediterranea* and *Cheiriphotis walkeri* especially in the; 1) fused carpus-propodus of male gnathopod 2; 2) propodus with transverse palm and; 3) uropod 3 uniramus. Further examination on the present species also indicated that *Cheiriphotis trifurcata* can be distinguished from *Cheiriphotis williamsoni* by the male gnathopod 1 which has the carpus longer than the propodus and the palm of male gnathopod 2 which bears 4 blunt teeth and 4 blunt teeth and one acute palmar corner. The present species also differs from *Cheiriphotis neotropicalis* in the carpus of male gnathopod 1 which is longer than the propodus and the propodus of male gnathopod 2 as long as broad in contrast to *Cheiriphotis neotropicalis* where the carpus of gnathopod 1 is subequal to propodus and propodus of gnathopod 2 is broader than long.

*Cheiriphotis trifurcata* shares a character of epimeron 2 with plumose setae on the ventral margin of epimera 2 with four known congeners, *Cheiriphotis erythraeus*, *Cheiriphotis mediterranea*, *Cheiriphotis williamsoni* and *Cheiriphotis neotropicalis*. The former can be distinguished from *Cheiriphotis erythraeus* by the carpus of the male gnathopod 1 which is partly fused with the propodus, the transverse palm which has 4 blunt teeth and the uniramus uropod while in the latter the carpus of the male gnathopod 1 is not fused with the propodus, the palm is medially V-shaped excavated with two teeth on both sides and the uropod is biramus. *Cheiriphotis trifurcata* is easily separated from *Cheiriphotis mediterranea* by the distally expanded peduncle of uropod 3 (vs. peduncle of uropod 3 not expanded distally).

To date, only one species of *Cheiriphotis* (i.e. *Cheiriphotis megacheles*)has been reported from the Andaman Sea and the South China Sea (Imbach 1967 and Rabindranath 1971). The absence of robust setae along the palm of gnathopod 1, the unfused carpus-propodus of the male gnathopod 2, a rounded epimeron 2, and the biramus uropod 3 in *Cheiriphotis megacheles* readily differentiates that species from the present one.

**Table 1.** Comparison of some distinguished characters between *Cheiriphotis trifurcata* sp. n. and the related species

Characters	Accessory flagellum	♂ G1 palm	♂ G1 carpus: propodus	♂G2 carpus and propodus	♂G2 propodus	♂ G2 palm	epimeron 2	♂pleopod 3	U3
*Cheiriphotis trifurcata*	4 articles	oblique with a robust seta at the proximal half	>	fused	as broad as long	palm transverse, with 5 blunt teeth	with plumose setae on ventral margin	outer ramus last article modified	uniramus peduncle expanded distally
*Cheiriphotis erythraeus* Ruffo,1969	4 articles	oblique, palm longer than posterior margin	=	not fused	longer than broad	palm transverse, half way excavated with two teeth on both side and a defining tooth	with plumose setae on ventral margin	normal	biramus, inner ramus small
*Cheiriphotis mediterranea* Myers, 1983	4 articles	oblique, posterodistal excavated	>	fused	broader than long	palm transverse, with 3 rounded lobes and a defining tooth	with plumose setae on ventral margin	normal	uniramus, peduncle poorly expanded
*Cheiriphotis megacheles (*Giles,1885)	5 articles	oblique, not distinctly defined,	>	not fused	as long as broad	palm oblique, palmar corner with a strong tooth projecting posteriorly	round	normal	biramus, inner ramus small
*Cheiriphotis neotropicalis* Valerio-Berado et al. 2007	3 articles, last article small	palm excavate, defining palm with subdistal robust seta	=	fused	broader than long	palm transverse, with three rounded lobes and a defining tooth	with plumose setae on ventral margin	normal	uniramus, peduncle poorly expanded
*Cheiriphotis walkeri* Stebbing, 1918	no data	palm oblique, emarginate at anterior margin	>	fused	broader than long	palm transverse with 2 depressions	round	normal	uniramus
*Cheiriphotis williamsoni* Salman & Jabbar, 1990	4 articles	palm oblique, longer than posterior margin	<	not fused	slightly longer than broad	palm transverse, with 3 large blunt teeth and a defining tooth,	round	normal	uniramus, peduncle broad

### World key to species of *Cheiriphotis*

**Table:** 

1	Male gnathopod 2 carpus vestigial, partly fused with propodus	2
–	Male gnathopod 2 carpus not fused with propodus	6
2	Male gnathopod 1 palm excavate, palmar corner with subdistal robust seta, carpus subequal to propodus	*Cheiriphotis neotropicalis* Valerio-Berardo, de Sousa & Rodigues, 2007
–	Male gnathopod 1 palm not excavate	3
3	Male gnathopod 1 carpus shorter than propodus, palm longer than hind margin	*Cheiriphotis williamsoni* Salman & Jabbar, 1990
–	Male gnathopod 1 capus longer than propodus, palm shorter than hind margin	4
4	Epimeron 2 without plumose setae on ventral margin, male gnathopod 2 basis dilated in anterodistal corner	*Cheiriphotis walkeri* Stebbing, 1918
–	Epimeron 2 with plumose setae on ventral margin, male gnathopod 2 basis not dilated in anterodistal corner	5
5	Male outer ramus of pleopod 3 tip modified into fork shape, gnathopod 2 carpus rectangular	*Cheiriphotis trifucata*sp. n.
–	Male outer ramus of pleopod 3 not modified, gnathopod 2 carpus triangular	*Cheiriphotis mediterranea* Myers, 1983
6	Accessory flagellum 2 articles	*Cheiriphotis minima* Ledoyer, 1982
–	Accessory flagellum more than 2 articles	7
7	Male gnathopod 2 palm oblique	8
–	Male gnathopod 2 palm transverse	14
8	Male gnathopod 1 palm acute, longer than hind margin	*Cheiriphotis australiae* Stebbing, 1910
–	Male gnathopod 2 palm not as above, not longer than hind margin	9
9	Uropod 3 uniramus	10
–	Uropod 3 biramus, inner ramus small	13
10	Male gnathopod 1 carpus subequal to propodus	*Cheiriphotis delloyei* Pirlot, 1934
–	Male gnathopod 2 carpus longer than propodus	11
11	Male gnathopod 2 propodus as long as broad	*Cheiriphotis megacheles* (Giles, 1885)
–	Male gnathopod 2 propodus longer than broad	12
12	Epimeron 2 with 2 notches on distoinferior corner	*Cheiriphotis pediformis* Myers, 1995
–	Epimeron 2 round on distoinferior corner	*Cheiriphotis madangensis* Ledoyer, 1979
13	Male gnathopod 2 basis robust, wider than half of the length	*Cheiriphotis durbanensis* K.H. Barnard, 1916
–	Male gnathopod 2 basic slender, not wider than half of the length	*Cheiriphotis rotui* Myers, 1989
14	Palm not excavated and smooth, defining tooth long like a finger, directed fore and upward	*Cheiriphotis quadrichelatus* Ortiz & Lalana,1997
–	Palm medially V-shaped excavated with two teeth on both sides and a not much longer defining tooth	*Cheiriphotis erythaeus* Ruffo, 1969

## Supplementary Material

XML Treatment for Cheiriphotis

XML Treatment for Cheiriphotis trifurcata
